# Interdialytic home systolic blood pressure variability increases all‐cause mortality in hemodialysis patients

**DOI:** 10.1002/clc.24259

**Published:** 2024-03-29

**Authors:** Liping Dong, Ming Tian, Hua Li, Junwu Dong, Xiaohong Song

**Affiliations:** ^1^ Department of Nephrology Wuhan Fourth Hospital Wuhan Hubei P.R. China; ^2^ Department of Clinical Nutrition Wuhan Fourth Hospital Wuhan Hubei P.R. China

**Keywords:** blood pressure variability, hemodialysis, home blood pressure measurement, mortality

## Abstract

**Background:**

The association between Interdialytic home blood pressure variability (BPV) and the prognosis of patients undergoing maintenance hemodialysis (MHD) largely unknown.

**Hypothesis:**

We proposed the hypothesis that interdialytic home BPV exert effect on cardiac and all‐cause mortality among individuals undergoing MHD.

**Methods:**

A total of 158 patients receiving MHD at the hemodialysis unit of Wuhan Fourth Hospital between December 2019 and August 2020 were included in this prospective cohort study. Patients were divided into tertiles according to the systolic BPV (SBPV), and the primary endpoints were cardiac and all‐cause death. Kaplan–Meier analysis was used to assess the relationship between long‐term survival and interdialytic home SBPV. In addition, Cox proportional hazards regression models were used to identify risk factors contributing to poor prognosis.

**Results:**

The risk of cardiac death and all‐cause death was gradually increased in patients according to tertiles of SBPV (3.5% vs. 14.8% vs. 19.2%, *p* for trend = .021; and 11.5% vs. 27.8% vs. 44.2%, *p* for trend <.001). The Cox regression analysis revealed that compared to Tertile 1, the hazard ratios for all‐cause mortality in Tertile 2 and Tertile 3 were 3.13 (*p* = .026) and 3.24 (*p* = .021), respectively, after adjustment for a series of covariates.

**Conclusions:**

The findings revealed a positive correlation between increased interdialytic home SBPV and elevated mortality risk in patients with MHD.

## Graphical abstract

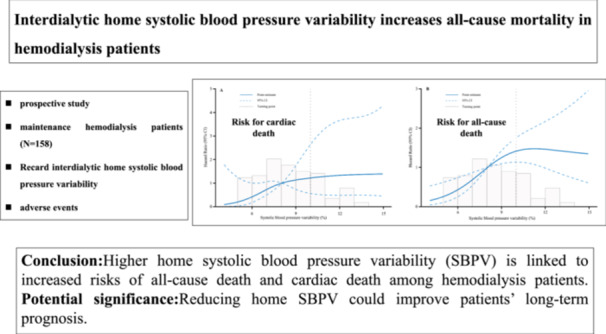

**Abbreviations**
ALB: albumin
AVF: arteriovenous fistulas
BPV: blood pressure variability
CKD: chronic kidney disease
CV: coefficient of variance
DBPV: diastolic blood pressure variability
ECW: extracellular water
ESRD: end‐stage kidney disease
MHD: maintenance hemodialysis
PA: phase angle
PTH: parathyroid hormone
SBPV: systolic blood pressure variability
TBW: total body water
TC: total cholesterol

## 1 BACKGROUND

The global prevalence of chronic kidney disease (CKD) is estimated to be 13.4%.^1^ End‐stage kidney disease (ESKD) is the most severe stage of disease progression, with approximately 3 million patients worldwide currently receiving dialysis. Over the past 20 years, the number of dialysis patients in developed nations has increased at an annual rate of 6%–12%, with even higher growth rates observed in developing countries, where hemodialysis is the primary form of kidney replacement therapy, accounting for more than 90%.^2^, ^3^ Furthermore, the prevalence of cardiovascular disease in hemodialysis patients exceeds 60%, accounting for more than 50% of deaths.^4^ Hypertension is common in hemodialysis patients with a prevalence of 90%.^5^ Elevated blood pressure can lead to endothelial dysfunction, atherosclerosis, and structural changes in the heart and brain, all of which increase the risk of death. In the general population, the risk of adverse cardiovascular events increases linearly with increasing systolic blood pressure.^6^ Nevertheless, the effect of blood pressure on the prognosis of patients with MHD is more complicated, as blood pressure can influence patient prognosis, irrespective of predialysis， interdialytic，or after dialysis, abnormal blood pressure levels, whether excessively high or low, are significantly correlated with an increased risk of cardiovascular and cerebrovascular adverse events, as well as all‐cause mortality.^7^ Despite well‐controlled mean blood pressure, high variability, and paroxysmal hypertension during visits still significantly increase the risk of cardiovascular adverse events, this illustrated that the prognosis of patients with MHD is not only dependent on the level of blood pressure but also correlated with BPV.^8^ Recently, BPV has been acknowledged as an independent cardiovascular risk factor in the general population and in patients with hypertension.^9^ For instance, Nardin and colleagues demonstrated that both short‐term and long‐term increased BPV are associated with an elevated risk of cardiovascular events and all‐cause mortality, this conclusion also applies to hemodialysis patients, as increased BPV during both the intradialytic and interdialytic measurements yield similar results.^10^, ^11^ However, the majority of current studies on BPV calculation in hemodialysis patients primarily depend on office and intradialytic blood pressure; home blood pressure monitoring is more repeatable and accurate and can truly reflect a patient's daily blood pressure fluctuations, moreover, it exhibits a stronger correlation with clinical adverse events.^12^, ^13^, ^14^ However, there exists a few reports on the association between interdialytic home BPV and the prognosis of patients undergoing MHD. Therefore, we conducted a prospective study to investigate the impact of interdialysis home BPV on the outcomes in MHD patients.

## 2 METHODS

### 2.1 Study design and participants

This single‐center prospective cohort study was designed to assess the impact of home BPV on the survival outcomes in patients with MHD. Patients with ESKD who underwent MHD in the hemodialysis unit of Wuhan Fourth Hospital from December 2019 to August 2020 were selected and informed consent was obtained. Inclusion criteria were (1) age ≥18 years, (2) dialysis vintage ≥3 months, (3) cooperation with home measurement of upper extremity blood pressure, and (4) undergoing bioelectrical impedance measurements to assess fluid load and other health indicators. Exclusion criteria were (1) patients with complicated with severe refractory heart failure (NYHA Class III–IV), (2) those who had experienced severe infection or acute cardiovascular and cerebrovascular accidents within 3 months before the study, (3) patients with severe liver disease or malignant tumor, and (4) individuals who underwent kidney transplantation or peritoneal dialysis during the follow‐up.

### 2.2 Study methods

Before the study, all patients underwent training to correctly measure blood pressure. All patients used a corrected electronic sphygmomanometer to measure brachial artery blood pressure; blood pressure was recorded four times a day for a week on a nondialysis day, before breakfast (7:00–8:30), before lunch (10:30–12:00), before dinner (17:00–19:00), and before going to bed. Patients taking antihypertensive medication were asked to measure their blood pressure before taking the medication. The BPV coefficient of variance (CV) was calculated as CV = (standard deviation/mean) × 100%. Both the systolic and diastolic BPV and mean blood pressure were calculated according to the blood pressure measured several times.

Bioelectrical impedance analysis‐based body composition analysis were used for assessing fuid volume and nutritional status in MHD patients. Body composition was performed using multifrequency electric bioimpedance (model InBody S10，Biospace Co., Ltd.) immediately after hemodialysis.

In addition, baseline data, including gender and age, dialysis vintage, vascular access, dialysis prescription, primary kidney disease, history of ischaemic heart disease was detected by reviewing medical records for confirmation or asking patients directly‐which included angina, myocardial infarction, or exist other evidence of ischaemic heart disease, such as coronary angiography results shows vascular stenosis, smoking history, hemoglobin, serum albumin (ALB), creatinine, urea nitrogen, triglycerides, total cholesterol (TC), parathyroid hormone (PTH), calcium, phosphorus, serum uric acid, and bioelectrical impedance, were collected.

### 2.3 Endpoints

The patients were followed using outpatient follow‐up, telephone communication, and in‐patient medical record inquiry. They were followed up until April 30, 2023. The endpoints were cardiac death (including myocardial infarction, malignant arrhythmia, and heart failure), all‐cause death, or termination of follow‐up. This study was approved by the Ethics Committee of the Wuhan Fourth Hospital (No. KY2022‐046‐01) in accordance with the Declaration of Helsinki and authorized by Medical Ethics.

### 2.4 Statistical analysis

Continuous variables conforming to the normal distribution are denoted by mean ± standard deviation (SD). Non‐normally distributed continuous variables are denoted by interquartile ranges (IQR), and counting data are expressed as frequency and percentage. Left ventricular ejection fraction (LVEF) is described with the minimum, maximum, and median values. The *t* test was used to compare the measurement data between two groups subjected to normal distribution, whereas the Mann–Whitney *U* or Kruskal–Wallis *H* test was used to compare between two or more groups without a normal distribution. The *χ* ^2^ test was used to compare the categorical data between groups. A restricted cubic spline risk curve was used to describe the association between elevated levels of systolic blood pressure variability (SBPV) and the risk of outcome events. Kaplan–Meier survival curves and Log‐rank tests were used to compare the differences in survival among tertiles. Univariate Cox regression analysis was used to identify variables affecting the occurrence of all‐cause mortality among patients, and subsequently, significant variables were included in multivariate Cox regression analysis to ascertain the effect of SBPV on cardiovascular death and all‐cause mortality. All statistical tests were performed by two‐sided test, and *p* < .05 was considered statistically significant. Statistical analyses were performed using R version 3.6.0 and SPSS 26.0. Photoshop version 6.0 were used for graphs.

## 3 RESULTS

### 3.1 Basic characteristics of patients

A total of 158 patients were included in the study, consisting of 95 males (60.1%) and 63 females (39.9%). The age of patients varied from 25 to 88 years old, with an average age of 57 ± 13 years old. The systolic blood pressure variation ranged from a minimum of 2.6% to a maximum of 20.1%, with a median value of 8.05%. The diastolic BPV (DBPV) ranged from a minimum of 2.6% to a maximum of 16.8%, with an average value of 7.63%. The primary causes of uremia included primary glomerulonephritis in 73 (46.2%) cases, diabetic nephropathy in 49 (31%) cases, hypertensive kidney lesion in 21 (13.3%) cases, renal allograft dysfunction in 5 (3.2%) cases, obstructive nephropathy in 4 (2.5%) cases, polycystic kidney in 4 (2.5%) cases, and lupus nephritis in 2 (1.3%) cases. We found that 146 patients (92.4%) had hypertension, 52 (32.9%) had diabetes, 33 (20.9%) had ischaemic heart disease, and 33 (20.9%) smoked. Arteriovenous fistulas (AVF) were selected for vascular access for hemodialysis in 117 (74.1%) patients, and tunneled cuffed catheter was selected in 41 (25.9%) patients. In total, 35 (22.2%) patients underwent hemodialysis twice a week, 21 (13.3%) patients five times every 2 weeks, and 102 (64.6%) patients received hemodialysis thrice a week. The shortest and longest dialysis vintages varied from 9 months to 207 months. The shortest follow‐up was for 4 months, the longest was for 40 months, and the median follow‐up was 33 months; among the patients, 44 deaths occurred during the follow‐up period. Twenty deaths were attributed to cardiovascular causes, including 1 who had sudden cardiac death, 10 died of congestive heart failure, and 9 who had ischemic heart disease.

### 3.2 Differences in clinical outcomes

We divided patients into survival and death groups and compared the baseline data. Compared with patients in the survival group, patients in the death group were older (67.5 [58.5, 75.5] vs. 56 [46, 63], *p* < .001). The proportion of male patients was higher (75% vs. 54.4%, *p* = .018), the percentage of ischemic heart disease was higher (31.8% vs. 16.7%, *p* = .036), ALB level was lower (36.8 ± 3.3 vs. 38.8 ± 3.2, *p* = .001), serum phosphorus was lower (1.59 ± 0.39 vs. 1.77 ± 0.44, *p* = .019). The variability (%) of SBP and DBP was higher (9.55 [7.83, 11.65] vs. 7.5 [5.28, 9.6], *p* < .001, 8.30 ± 2.41 vs. 7.38 ± 2.57, *p* = .041). The proportion of diabetic patients was higher (54.5% vs. 24.6%, *p* < .001). Bioelectrical impedance measurements showed that patients in the dead group had a higher extracellular water (ECW) to total body water (TBW) (0.40 ± 0.01 vs. 0.39 ± 0.01, *p* < .001) and a smaller phase angle (PA) (4.65 ± 1.12 vs. 5.44 ± 1.06, *p* < .001). No significant difference was found between the two groups in terms of dialysis vintage, dialysis prescription, lipid, calcium, hyperparathyroidism (PTH), and medication history (Supporting Information S1: Table 3). To further illustrate the baseline characteristics of patients, we compared them based on cardiac and without cardiac deaths (Supporting Information S1: Table 4).

### 3.3 Relationship between BPV and patient outcomes

**Figure 1:**
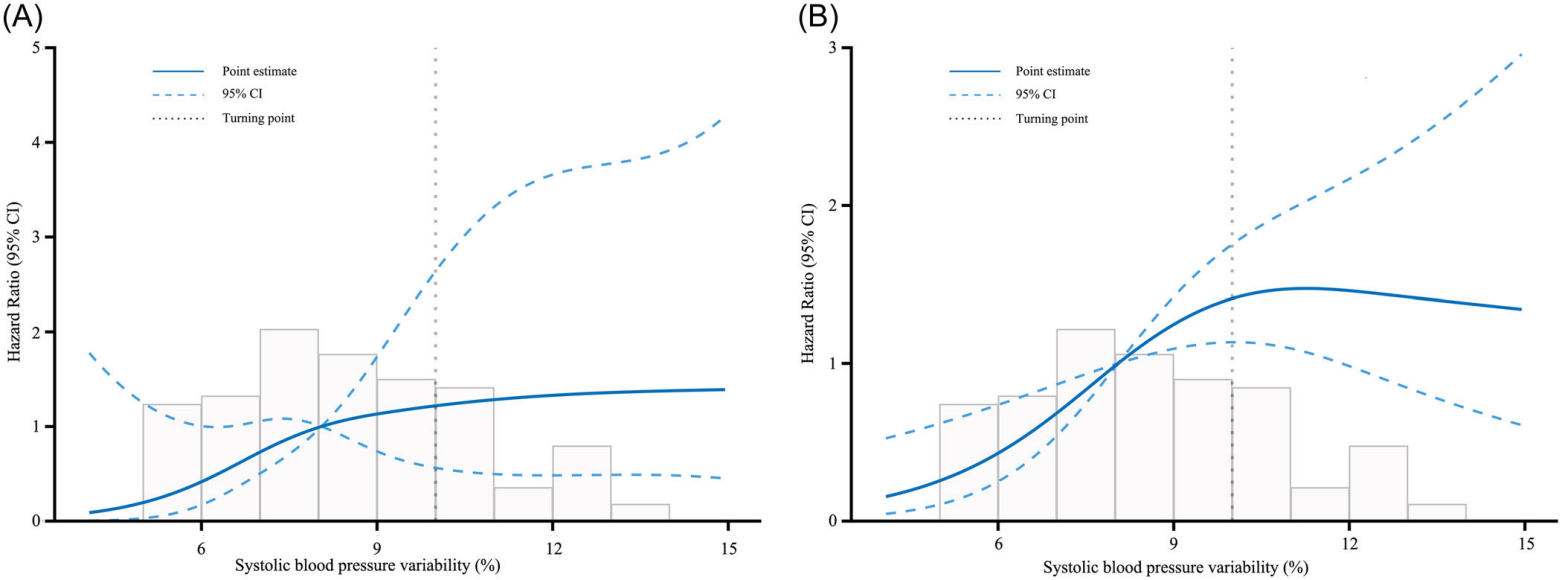
Restricted cubic spline curves of the risk for cardiovascular mortality (A) and all‐cause death (B).

**Figure 2:**
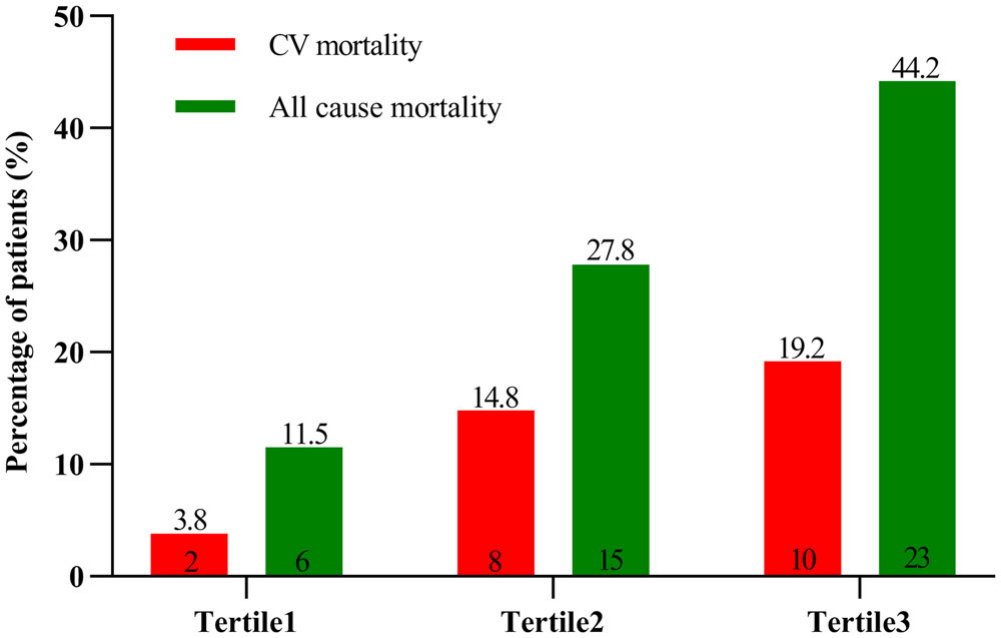
Percentage of cardiovascular and all‐cause mortality in tertiles of interdialysis home systolic blood pressure variability.

**Figure 3:**
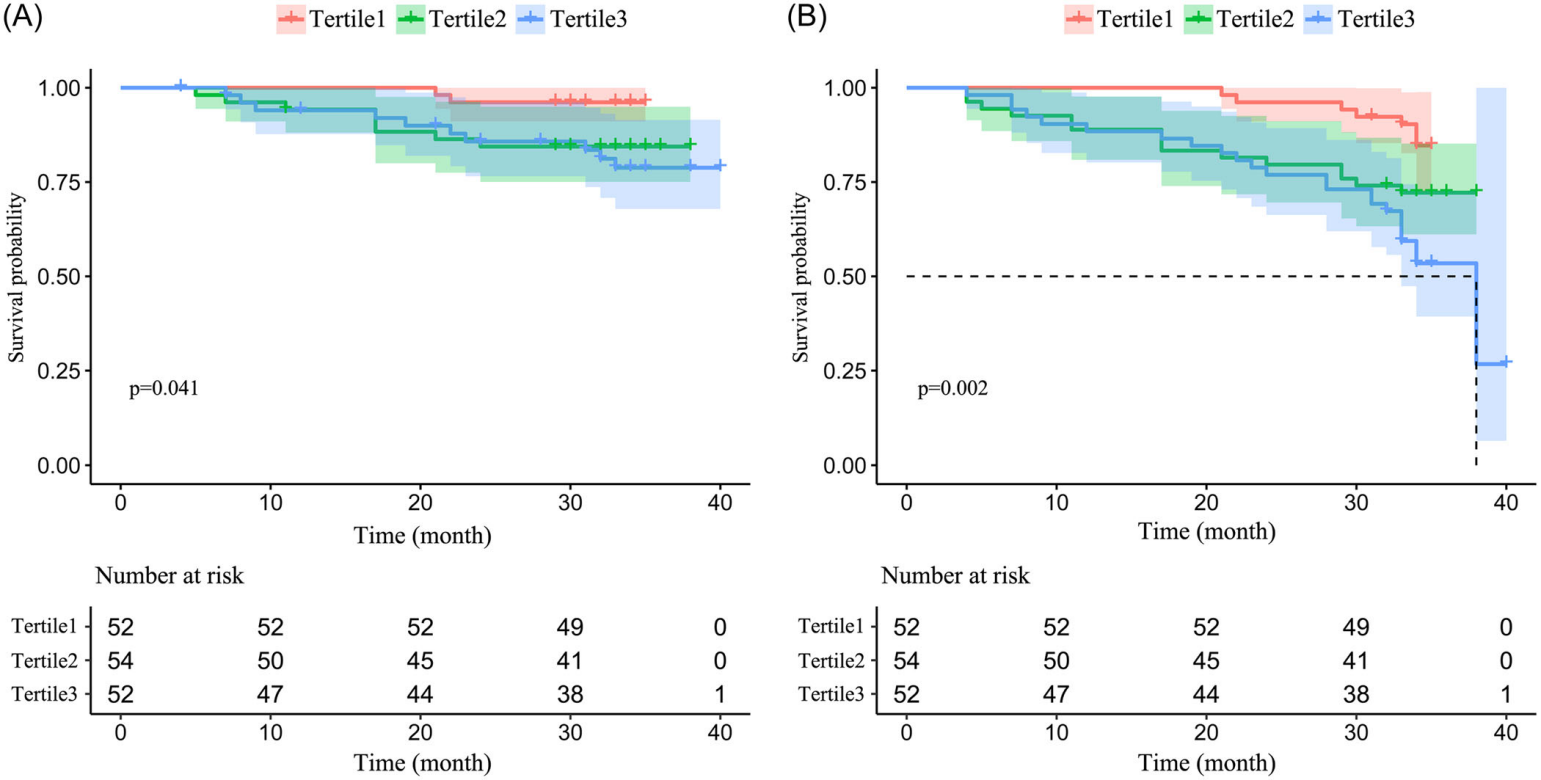
Kaplan–Meier survival curves and life tables for occurrence of the endpoint (A) cardiovascular mortality and all‐cause death (B).

To assess the effect of BPV on long‐term outcomes, a restricted cubic spline model was employed to analyze the data. The results revealed that an increase in SBPV within a specific range was associated with a higher risk of cardiac death and all‐cause death (Figure 1A,B). Conversely, no similar trend was observed for DBPV. Therefore, patients were divided into tertiles according to the SBPV, and significant differences were observed among tertiles when comparing the baseline data (Table 1). Compared with Tertile 1, both Tertile 2 and Tertile 3 were older (60.5 [55, 70] vs. 60.5 [48, 65] vs. 54.5 [45, 64], *p* = .025), the proportion of diabetic patients was higher (46.2% vs. 35.2% vs. 17.3%, *p* = .007), DBPV (%) was greater (9.36 ± 2.41 vs. 7.51 ± 1.92 vs. 6.04 ± 2.18, *p* < .001). In addition, significant differences among tertiles in the results of bioelectrical impedance, including higher extracellular fluid content and body fat content (PBF: 28.27 ± 9.29 vs. 27.65 ± 10.50 vs. 22.54 ± 9.68, *p* = .006; VFA: 75 [57.53, 106.85] vs. 79.15 [44.9, 118.63] vs. 48.05 [34.05, 90.88], *p* = .003), and lower muscle mass (39.7 [33, 45.3] vs. 39.3 [34.8, 48.7] vs. 44.3 [38.8, 49.2], *p* = .04), and significant decrease in PA (4.84 ± 1.12 vs. 5.37 ± 1.11 vs. 5.45 ± 1.08, *p* = .01) in Tertile 2 and Tertile 3 compared to Tertile 1. In terms of clinical outcomes, patients in Tertile 2 and Tertile 3 exhibited a progressive increase in both cardiac death and all‐cause death when compared to those in Tertile 1 (3.5% vs. 14.8% vs. 19.2%, *p* for trend = .021, and 11.5% vs. 27.8% vs. 44.2%, *p* for trend <.001) (Figure 2). The Kaplan–Meier survival analysis demonstrated a significant difference in cumulative survival between Tertile 1, Tertile 2, and Tertile 3, and the higher SBPV associated with an increased risk of cardiac death (Log‐rank test *χ* ^2^ = 6.372, *p* = .041) and all‐cause death (Log‐rank test *χ* ^2^ = 12.677, *p* = .002) (Figure 3A,B).

**Table 1:** Baseline characteristics of study population by tertiles of interdialysis home systolic blood pressure variability.

Characteristics	SBPV‐tertile1	SBPV‐tertile2	SBPV‐tertile3	*p* Value
	2.60–6.70	6.80–9.40	9.50–20.10	
	*N* = 52	*N* = 54	*N* = 52	
Gender, female, *N* (%)	18(34.6)	23(42.6)	22(42.3)	.639
Age(years)	54.5[45,64]	60.5[48,65]	60.5[55,70]	.025
Dialysis vintage (months)	58[42.3,92.3]	56.5[44.8,87.3]	62[48,79.8]	.832
Vascular access, *N* (%)				.305
Tunneled cuffed catheter	11(21.2)	18(33.3)	12(23.1)	
Avf	41(78.8)	36(66.7)	40(76.9)	
Dialysis prescription, *N* (%)				.071
Twice a week	16(30.8)	9(16.7)	10(19.2)	
Five times every 2 weeks	7(13.5)	11(20.4)	3(5.8)	
Three times a week	29(55.8)	34(63.0)	39(75.0)	
Hypertension, *N* (%)	47(90.4)	52(96.3)	47(90.4)	.372
Diabetes, *N* (%)	9(17.3)	19(35.2)	24(46.2)	.007
History of smoking, *N* (%)	12(23.1)	11(20.4)	10(19.2)	.884
Ischemic heart disease, *N* (%)	9(17.3)	10(18.5)	14(26.9)	.420
LVEF,%	60(49–65)	60(32–65)	58(30–63)	.220
Hemoglobin (g/L)	107[93,117]	105[95,112]	110[94,118]	.748
Albumin (g/L)	39.4 ± 3.7	38.0 ± 2.6	37.4 ± 3.4	.007
Urea nitrogen (mmol/L)	22.0 ± 6.6	23.2 ± 5.6	23.9 ± 6.2	.303
Creatinine (umol/L)	919.1 ± 226.3	947.5 ± 235.1	824.3 ± 292.4	.035
Phosphorus (mmol/L)	1.71 ± 0.46	1.71 ± 0.37	1.73 ± 0.47	.981
Calcium (mmol/L)	2.12[1.95,2.26]	2.15[2.00,2.29]	2.06[1.90,2.26]	.43
PTH (pg/mL)	363[189,587]	425[299,643]	398[228,700]	.181
Uric acid (umol/L)	439[364,509]	452[384,488]	434[346,550]	.972
Triglyceride (mmol/L)	1.27[0.85,2.14]	1.32[1.05,1.66]	1.25[0.97,1.86]	.953
TC (mmol/L)	3.61[3.16,4.45]	3.67[3.23,4.34]	3.55[2.86,4.56]	.681
LDL (mmol/L)	1.90 ± 0.65	2.18 ± 0.67	2.09 ± 0.78	.156
systolic BP(mmHg), mean ± SD	141 ± 17	141 ± 14	138 ± 17	.503
diastolic BP(mmHg), mean ± SD	83 ± 10	80 ± 10	76 ± 10	.003
pulse pressure(mmHg), mean ± SD	59 ± 15	62 ± 15	62 ± 16	.547
SBPV (%)	4.95[4.3,5.98]	8.05[7.48,8.70]	11.35[10.2,13.23]	<.001
DBPV (%)	6.04 ± 2.18	7.51 ± 1.92	9.36 ± 2.41	<.001
Antihypertensive drug, *N* (%)				
ACEI	21(40.4)	21(38.9)	20(38.5)	.978
ARB	26(50.0)	31(57.4)	26(50.0)	.676
β‐blocker	45(86.5)	49(90.7)	45(86.5)	.743
CCB	47(90.4)	49(90.7)	45(86.5)	.743
α‐blockers	19(36.5)	16(29.6)	16(30.8)	.719
BMI (kg/m^2^)	22.24 ± 2.91	22.44 ± 3.54	21.93 ± 3.30	.724
ICW (L)	21.10 ± 3.99	19.94 ± 3.91	18.96 ± 4.05	.025
TBW (L)	34.44 ± 6.42	32.51 ± 6.15	31.39 ± 6.52	.049
ECW (L)	13.15[12,15.4]	12[10.6,14.6]	12.45[10.23,13.88]	.151
ECW/TBW	0.39 ± 0.01	0.39 ± 0.01	0.40 ± 0.01	.001
PBF (%)	22.54 ± 9.68	27.65 ± 10.50	28.27 ± 9.29	.006
SLM (kg)	44.3[38.8,49.2]	39.3[34.8,48.7]	39.7[33,45.3]	.040
WC (cm)	73.8[65.83,87.15]	81[73,92.55]	80.25[72.03,86.83]	.039
VFA (cm^2^)	48.05[34.05,90.88]	79.15[44.9,118.63]	75[57.53,106.85]	.003
PA	5.45 ± 1.08	5.37 ± 1.11	4.84 ± 1.12	.010

*Note*: Values are expressed as mean ± standard deviation [SD] or median [interquartile range [IQR], 25th–75th percentile].

Abbreviations: ACEI, angiotensin converting enzyme inhibit; ARB, angiotensin receptor blocker; BMI, body mass index; DBPV, dystolic blood pressure variability; ECW, extracellular water; ICW, intracellular water; LDL, low‐density lipoprotein; LVEF, left ventricular ejection fraction; N, number of individuals; PA, phase angle; PBF, percentage of body fat; PTH, parathyroid hormone; SBPV, systolic blood pres‐sure variability; SLM, soft lean mass; TBW, total body water; TC, triglyceride; VFA, visceral fat area; WC, waist circumference.

As age increases, BPV increases accordingly. To compare the possible effects of BPV in different age groups, we grouped patients at the age of 60‐year‐old for comparison (Supporting Information S1: Table 5). Univariate Cox regression analysis revealed that gender, serum albumin, serum phosphorus, diastolic BP, pulse pressure, DBPV, age, diabetes, ECW/TBW, and PA were associated with death (Supporting Information S1: Table 6). Furthermore, patients in Tertile 2 and Tertile 3 exhibited a significantly higher risk of cardiac death and all‐cause death compared to those in Tertile 1 (*p* < .05). Thus, after adjusting for different variables and building different models, we found that the risk of all‐cause death was still significantly higher in Tertile 2 and Tertile 3 compared with Tertile 1. Although this trend was not significant in the risk of cardiac death, we found that the SBPV increased with an increase in the risk of death (Table 2).

**Table 2:** Associations between tertiles of interdialysis home systolic blood pressure variability and all‐cause and cardiovascular (CV) mortality.

outcome	No. events of *N*	Univariable analysis		Multivariable analysis‐Model 1		Multivariable analysis‐Model 2		Multivariable analysis‐Model 3	
		HR[95% CI]	*p* Value	HR[95% CI]	*p* Value	HR[95% CI]	*p* Value	HR[95% CI]	*p* Value
CV mortality	20 of 158								
SBPV‐tertile1	2 of 52	1	Ref.	1	Ref.	1	Ref.	1	Ref.
SBPV‐tertile2	8 of 54	4.43[0.94–20.85]	0.060	4.15[0.88–19.65]	0.073	6.25[1.15–33.88]	0.034	5.58[1.02–30.53]	0.047
SBPV‐tertile3	10 of 52	5.76[1.26–26.31]	0.024	3.30[0.72–15.27]	0.126	3.53[0.69–18.08]	0.131	4.72[0.85–26.16]	0.076
All cause mortality	44 of 158								
SBPV‐tertile1	6 of 52	1	Ref.	1	Ref.	1	Ref.	1	Ref.
SBPV‐tertile2	15 of 54	2.76[1.07–7.11]	0.036	2.44[0.94–6.32]	0.066	3.24[1.19–8.79]	0.021	3.13[1.15–8.52]	0.026
SBPV‐tertile3	23 of 52	4.47[1.82–11.01]	0.001	2.75[1.10–6.86]	0.030	3.18[1.20–8.43]	0.020	3.24[1.20–8.74]	0.021

*Note*: Hazard ratio ratios (HR) and 95% confidence intervals (CIs) were obtained by univariate Cox regression analysis and multivariate Cox regression analysis. Modle1, adjustment for age and diabetes. Modle2, adjustment for age, gender, diabetes, Albumin, dystolic blood pressure variability and phosphorus. Modle3, adjustment for age, gender, diabetes, Albumin, dystolic blood pressure variability, phosphorus, ECW/TBW, phase angle, diastolic BP, pulse pressure.

Abbreviations: DBPV, dystolic blood pressure variability; ECW, extracellular water; SBPV, systolic blood pres‐sure variability; TBW, total body water.

## 4 DISCUSSION

In this prospective cohort study, the prevalence of hypertension among patients with MHD was found to be 92.4%, significantly higher than that in the general population. The risk of cardiac and all‐cause mortality was significantly higher in patients with high home SBPV, this result was similar to those of previous studies,^15^ while the present study found there is no U‐shaped curve relationship between home SBPV and adverse outcomes. Previous researchers have demonstrated that both short‐term and long‐term SBPV are risk factors for poor outcomes in patients with MHD, whereas conclusions about the effect of DBPV on patient survival outcomes are not entirely consistent.^16^, ^17^, ^18^, ^19^

Undergoing hemodialysis two to three times a week leads to significant fluctuations in blood pressure in MHD patients. The KDOQI and the Canadian Society of Nephrology recommend using pre‐ and postdialysis BP measurements to guide hypertension management; however, studies have found a stronger correlation between home blood pressure and poor prognosis in MHD patients,^20^, ^21^, ^22^ but no clear conclusion about effect of home BPV on MHD patients prognosis has been reached. The majority of current studies on BPV calculation in hemodialysis patients primarily depend on office and intradialytic blood pressure; our present prospective study has clarified the association between home BPV and poor prognosis.

BPV reflects the degree of fluctuation in blood pressure over time. BPV can be divided into short‐term, medium‐term, and long‐term variations. Short‐term variation usually refers to BPV within 24 h, which can be divided into BPV during the cardiac cycle, BPV within a few minutes or hours, and circadian rhythm changes. In contrast, long‐term BP variability primarily includes day‐to‐day, visit‐to‐visit, or seasonal BP variability. Compared with short‐term BPV, long‐term BPV has better reproducibility and less random variability.^23^, ^24^ In the present study, we selected interdialytic home BPV for a week on nondialysis days belongs to long‐term BPV. Currently, there exists no widely accepted measure of BPV, which is usually expressed as standard deviation, coefficient of variation, average real variability (ARV), and variation independent of the mean (VIM). Compared with the standard deviation, the coefficient of variation can overcome the effect of mean blood pressure on BPV, does not consider the sequence of blood pressure measurements, and is easy to calculate clinically. Although VIM and ARV can respond to fluctuations independent of mean BP, the calculations are complex and difficult to generalize in clinical practice. Rossignol and colleagues reported that the CV of blood pressure was the most appropriate to measure BPV in patients with MHD.^25^ In addition, long‐term monitoring of BPV depends on accurate, repeatable blood pressure measurements. Home blood pressure monitoring can provide multiple blood pressure readings in an individual's daily environment, avoiding white coats and masked hypertension. Moreover, the affordable, long‐term use of the method is easily accepted by patients and is the best method for long‐term follow‐up of patients with hypertension.^13^, ^26^ Therefore, this study demonstrated that home blood pressure CV can better predict long‐term adverse events in patients.

Elevated BPV has been linked to several pathological conditions, including peripheral artery disease, cardiovascular disease, and diabetes, as well as factors such as older age, female gender, increased arterial stiffness, autonomic nervous dysfunction, low body mass index, excessive alcohol consumption, dietary habits, smoking, and sedentary lifestyles.^27^, ^28^, ^29^ In hemodialysis patients, several diseases often coexist, and the body is in a state of chronic micro‐inflammation for a prolonged time. Although the metabolic substances, vasoactive mediators, and water accumulated during hemodialysis are removed, severe changes in hemodynamics and internal environment in a short time cause significant fluctuations in blood pressure. Consistent with previous studies, we found higher BPV in older patients with diabetes.^30^ In addition, we found that outcomes derived from bioelectrical impedance revealed a correlation between increased visceral fat content and body fat percentage, diminished muscle content and phase angle, and heightened BPV. Obesity or visceral obesity has been found to potentially contribute to the upregulation of adipocytokines, and heightened inflammatory activity, ultimately leading to diminished insulin sensitivity, dyslipidemia, endothelial dysfunction, development of atherosclerosis, and increased stiffness.^31^, ^32^, ^33^ Reduced muscle tissue can lead to insulin resistance, endothelial dysfunction, inflammation, oxidative stress, activation of the renin–angiotensin–aldosterone system (RAAS), and autophagy dysregulation in adipocytes.^34^, ^35^, ^36^ Kato and colleagues reported a significant negative correlation between the thigh muscle area and carotid intima‐media thickness, cardio‐malleolus vascular index, and ankle‐brachial index in patients with MHD.^37^ Previous studies have demonstrated an independent association between PA and nutritional and inflammatory markers in patients with uremia, with a smaller PA, indicating a higher severity of atherosclerosis and vascular calcification.^38^

Compared to average blood pressure, BPV exerts a more pronounced impact on the damage inflicted by hypertension on target organs. Elevated BPV signifies greater fluctuations and consequently a heightened susceptibility to cardiovascular and cerebrovascular diseases.^39^ A meta‐analysis revealed that with each 1 mmHg increase in the standard deviation of systolic blood pressure, the likelihood of all‐cause mortality increased by 3%, cardiovascular death by 10%, and stroke by 2%.^40^ The mechanism underlying blood pressure fluctuations contributing to target organ damage and adverse events remains elusive; it could be associated with increased arterial stiffness, remodeling of the microcirculation, inflammatory response, activation of the sympathetic nervous system, and RASS, and so forth.^41^ Our findings indicated that, when SBPV was less than 10%, there was a gradual increase in the risk of cardiac death and all‐cause death as the variability increased. However, when the SBPV exceeded 10%, the relationship between SBPV and adverse events became less apparent. Also, we found that the risk of all‐cause death was higher in Tertile 3 than in Tertile 2 (3.24 vs. 3.13), this trend was not observed in cardiac death (5.58 vs. 4.72). We think this unexpected finding may be due to the small sample size and short follow‐up time, and further investigation with long‐term follow‐up is warranted to confirm these findings.

Additional reductions in blood pressure variability are beneficial in improving patient outcomes. Limited evidence suggests that the administration of calcium channel blockers, controlled fluid intake, and decreased ultrafiltration volume could contribute to the reduction of BPV in patients with MHD. Conversely, the use of antihypertensive regimens containing β‐blockers and RAAS system inhibitors is not beneficial in improving interdialysis BPV in dialysis patients.^18^, ^19^, ^42^

The study had certain limitations. First, home blood pressure measurement could not evaluate the fluctuations in blood pressure during sleep; we were unable to compare predialysis BP variability, postdialysis BP variability, and home BP variability simultaneously. Second, although we analyzed the effect of antihypertensive drug use on outcomes in each group, we were unable to assess compliance; we are also unable to accurately evaluate the impact of other factors on blood pressure variability. Third, the trend of home BP variability on cardiac death is not particularly significant, long follow‐up duration, more multicenter and large sample‐size studies are warranted to confirm these findings. Lastly, our study solely focused on assessing the correlation between the coefficient of variation and adverse outcome events, without analyzing the effects of other measures such as standard deviation, ARV, and VIM.

In conclusion, our findings suggest that higher home SBPV is linked to increased risks of all‐cause death and cardiac death among hemodialysis patients. Reducing home SBPV could improve patients' long‐term prognosis.

## CONFLICT OF INTEREST STATEMENT

The authors declare no conflict of interest.

## Supporting information

Supporting information.

## Data Availability

The data that support the findings of this study are available from the corresponding author upon reasonable request.
