# Supplementary material for: Interdialytic home systolic blood pressure variability increases all‐cause mortality in hemodialysis patients
Source: Clin Cardiol. 2024 Mar 29;47(4):e24259. doi: 10.1002/clc.24259 (PMC10979187; doi:10.1002/clc.24259)
Supplement: Supplementary file 1 — Supporting information. [file CLC-47-e24259-s001.docx]

**Supplementary Material**

| **Table 3.Baseline demographic, clinical and routine laboratory characteristics of the study participants.** | | | | | | |
| --- | --- | --- | --- | --- | --- | --- |
| **Characteristics** | | Total | Survival -group | Death-group | *P* | |
|  |  | 158 | 114 | 44 |  |  |
| Gender，Female , N (%) | 63 (39.9) | | 52 (45.6) | 11 (25) | 0.018 | |
| Age (years) | 59 [49,66] | | 56 [46,63] | 67.5 [58.5,75.5] | <0.001 | |
| Dialysis vintage (months), | 59 [44,82.8] | | 60.5 [46.8,82.8] | 53.5 [40,88.3] | 0.112 | |
| Vascular access, N (%) |  | |  |  | 0.522 | |
| Tunneled cuffed catheter | 41 (25.9) | | 28(24.6) | 13(29.5) |  | |
| Avf | 117 (74.1) | | 86(75.4) | 31(70.5) |  | |
| Dialysis prescription, N(%) |  | |  |  | 0.354 | |
| Twice a week | 35 (22.2) | | 22(19.3) | 13(29.5) |  | |
| Five times every 2 weeks | 21 (13.3) | | 15(13.2) | 6(13.6) |  | |
| Three times a week | 102 (64.6) | | 77(67.5) | 25(56.8) |  | |
| Hypertension，N (%) | 146 (92.4) | | 106(93.0) | 40(90.9) | 0.916 | |
| Diabetes，N (%) | 52 (32.9) | | 28(24.6) | 24(54.5) | <0.001 | |
| History of smoking，N (%) | 33 (20.9) | | 22(19.3) | 11(25.0) | 0.429 | |
| Ischemic heart disease,N (%) | 33 (20.9) | | 19(16.7) | 14(31.8) | 0.036 | |
| LVEF,% | 60(30-65) | | 58(30-63) | 60(36-65) | 0.081 | |
| Hemoglobin (g/L) | 107 [94,117] | | 107[94，117] | 108[93,117] | 0.892 | |
| Albumin (g/L) | 38.2±3.3 | | 38.8±3.2 | 36.8±3.3 | 0.001 | |
| Urea nitrogen (mmol/L), | 23.0±6.1 | | 23.1±6.4 | 22.8±5.5 | 0.745 | |
| Creatinine (umol/L) | 897.6±256.6 | | 921.7±241.6 | 835.3±285.7 | 0.058 | |
| Phosphorus (mmol/L) | 1.72±0.43 | | 1.77±0.44 | 1.59±0.39 | 0.019 | |
| Calcium (mmol/L) | 2.12 [1.97,2.28] | | 2.13[1.97,2.28] | 2.09[1.95,2.28] | 0.622 | |
| PTH (pg/ml) | 397 [264,604] | | 411[279，645] | 353[206，580] | 0.241 | |
| Uric acid (umol/L) | 444 [358,514] | | 444[357,510] | 443[360,518] | 0.984 | |
| Triglyceride (mmol/L) | 1.28 [0.98,1.94] | | 1.28[0.96,2.03] | 1.29[1.00,1.76] | 0.817 | |
| TC (mmol/L) | 3.63 [3.08,4.41] | | 3.67[3.25,4.52] | 3.32[2.70,4.36] | 0.069 | |
| LDL(mmol/L) | 2.06±0.71 | | 2.10±0.68 | 1.94±0.76 | 0.25 | |
| systolic BP(mmHg),  mean±SD | 140±16 | | 139±17 | 142±15 | 0.200 | |
| diastolic BP(mmHg),  mean±SD | 79±10 | | 81±10 | 76±9 | 0.022 | |
| pulse pressure(mmHg),  mean±SD | 61±15 | | 59±16 | 66±13 | 0.004 | |
| SBPV (%) | 8.05 (5.98,10.2] | | 7.5(5.28,9.6] | 9.55(7.83,11.7] | <0.001 | |
| DBPV (%) | 7.63±2.56 | | 7.38±2.57 | 8.30±2.41 | 0.041 | |
| Antihypertensive drug, N(%) |  | |  |  |  | |
| ACEI | 62 (39.2) | | 48(42.1) | 14(31.8) | 0.235 | |
| ARB | 83 (52.5) | | 57(50.0) | 26(59.1) | 0.305 | |
| β-blocker | 139 (88.0) | | 100(87.7) | 39(88.6) | 0.874 | |
| CCB | 141 (89.2) | | 102(89.5) | 39(88.6) | 0.879 | |
| α-blockers | 51 (32.3) | | 34(29.8) | 17(38.6) | 0.288 | |
| BMI (kg/m^2^) | 22.21±3.25 | | 22.26±3.32 | 22.07±3.10 | 0.749 | |
| ICW (L) | 20.00±4.05 | | 20.10±4.19 | 19.73±3.68 | 0.61 | |
| TBW (L) | 32.78±6.44 | | 32.78±6.61 | 32.77±6.05 | 0.995 | |
| PBF (%) | 26.17±10.11 | | 25.86±10.06 | 26.98±10.32 | 0.532 | |
| ECW/TBW | 0.39±0.01 | | 0.39±0.01 | 0.40±0.01 | <0.001 | |
| PA | 5.22±1.13 | | 5.44±1.06 | 4.65±1.12 | <0.001 | |
| ECW(L) | 12.75[10.60,14.70] | | 12.35[10.6,14.73] | 13.2[11,14.65] | 0.436 | |
| SLM(kg) | | 41.55[35.20,48.23] | 41.35[35.13,48.53] | 42.7[35.53,46.5] | 0.93 | |
| WC(cm) | | 79.20[71.10,89.00] | 79[70.38,89] | 79.6[72.13,90.33] | 0.777 | |
| VFA(cm2) | | 72.20[45.03,105.55] | 70[41.78，105.55] | 73.5[48.85,108.03] | 0.191 | |
| Values are expressed as mean±[Standard Deviation](https://blog.csdn.net/soga235/article/details/114546691) [SD] or median [[interquartile range [IQR]](https://www.researchgate.net/figure/Figure-Box-plots-of-interquartile-range-IQR-range-and-median-fluoride-concentrations_fig1_51530471), 25th-75th percentile], N, number of individuals; LVEF: left ventricular ejection fraction;PTH: parathyroid hormone；TC: triglyceride ; LDL: low‑density lipoprotein; SBPV: systolic blood pres-sure variability; DBPV: dystolic blood pressure variability; ACEI: angiotensin converting enzyme inhibit; ARB: angiotensin receptor blocker; BMI: Body Mass Index; ICW:Intracellular Water; TBW: Total Body Water; ECW:Extracellular Water ；PBF:Percentage of Body Fat；SLM:Soft Lean Mass；WC: Waist Circumference ；VFA: Visceral Fat Area；PA:Phase Angle. | | | | | |  |

| **Table 4. Baseline characteristics of study population by CV mortality.** | | | | |
| --- | --- | --- | --- | --- |
| **Characteristics** | Total | CV Mortality-group | nonCV Mortality -group | P |
|  | 158 | 20 | 138 |  |
| Gender，Female, N（%） | 63 (39.9) | 7 (35) | 56 (40.6) | 0.634 |
| Age(years) , median(IQR) | 59(49,66) | 72(60.8,79.3) | 57(48,65) | <0.001 |
| Dialysis vintage(months), median(IQR) | 59(44,82.8) | 52(38.3,76.5) | 60(44.8,85.3) | 0.221 |
| Vascular access,N(%) | 158 |  |  | 0.002 |
| Tunneled cuffed catheter | 41 (25.9) | 11 (55) | 30 (21.7) |  |
| Avf | 117 (74.1) | 9 (45) | 108 (78.3) |  |
| Dialysis prescription,N(%) | 158 |  |  | 0.868 |
| Twice a week | 35 (22.2) | 5 (25) | 30 (21.7) |  |
| Five times every 2 weeks | 21 (13.3) | 2 (10) | 19 (13.8) |  |
| Three times a week | 102 (64.6) | 13 (65) | 89 (64.5) |  |
| Hypertension，N（%） | 146 (92.4) | 20 (100) | 126 (91.3) | 0.357 |
| Diabetes，N（%） | 52 (32.9) | 11 (55) | 41 (29.7) | 0.024 |
| History of smoking，N（%） | 33 (20.9) | 5 (25) | 28 (20.3) | 0.628 |
| Ischemic heart disease, N (%) | 33 (20.9) | 9(45) | 24(17.4) | 0.005 |
| LVEF,% | 60(30-65) | 58(32-62) | 60(30-65) | 0.136 |
| Hemoglobin(g/L),median(IQR) | 107(94,117) | 108(92,115) | 107(94,117) | 0.724 |
| Albumin(g/L),mean±SD | 38.25±3.33 | 36.7±3.0 | 38.5±3.3 | 0.022 |
| Urea nitrogen(mmol/L), mean±SD | 23.0±6.1 | 22.50±5.20 | 23.11±6.27 | 0.681 |
| Creatinine(umol/L),mean±SD | 897.6±256.6 | 731.3±221.6 | 921.7±253.1 | 0.002 |
| Phosphorus(mmol/L),mean±SD | 1.7±0.4 | 1.51±0.24 | 1.75±0.45 | 0.001 |
| Calcium(mmol/L),median(IQR) | 2.12(1.97,2.28) | 2.17(2.04,2.28) | 2.12(1.97,2.28) | 0.469 |
| PTH(pg/ml), median(IQR) | 397(264,604) | 454(153,605) | 393(265,605) | 0.867 |
| Uric acid(umol/L), median(IQR) | 444(358,514) | 437(381,498) | 444(356,518) | 0.580 |
| Triglyceride (mmol/L), median (IQR) | 1.28(0.98,1.94) | 1.18(0.94,1.68) | 1.31(0.98,1.99) | 0.406 |
| TC (mmol/L), median (IQR) | 3.63(3.08,4.41) | 3.89(2.91,4.42) | 3.61(3.11,4.42) | 0.947 |
| LDL(mmol/L),mean±SD | 2.06±0.71 | 2.11±0.75 | 2.05±0.70 | 0.748 |
| systolic BP(mmHg),mean±SD | 140±16 | 146±14 | 139±16 | 0.112 |
| diastolic BP(mmHg),mean±SD | 79±10 | 78±9 | 80±10 | 0.431 |
| pulse pressure(mmHg),mean±SD | 61±15 | 68±14 | 60±15 | 0.029 |
| SBPV(%),median (IQR) | 8.05(5.98,10.20) | 9.20(8.35,11.73) | 7.80(5.88,10.13) | 0.016 |
| DBPV(%),mean±SD | 7.63±2.56 | 8.56±2.63 | 7.50±2.53 | 0.083 |
| Antihypertensive drug,N(%) |  |  |  |  |
| ACEI | 62 (39.2) | 5 (25) | 57 (41.3) | 0.163 |
| ARB | 83 (52.5) | 15 (75) | 68 (49.3) | 0.031 |
| β-blocker | 139 (88) | 20 (100) | 119 (86.2) | 0.161 |
| CCB | 141 (89.2) | 19 (95) | 122 (88.4) | 0.615 |
| α-blockers | 51 (32.3) | 8 (40) | 43 (31.2) | 0.429 |
| BMI(kg/m^2^) ,mean±SD | 22.21±3.25 | 22.60±3.63 | 22.15±3.20 | 0.569 |
| ICW(L) ,mean±SD | 20.00±4.05 | 18.14±3.24 | 20.27±4.09 | 0.028 |
| TBW(L) ,mean±SD | 32.78±6.44 | 30.38±5.67 | 33.12±6.49 | 0.075 |
| PBF（%）,mean±SD | 26.17±10.11 | 31.10±12.59 | 25.46±9.54 | 0.019 |
| ECW/TBW,mean±SD | 0.39±0.01 | 0.40±0.01 | 0.39±0.01 | <0.001 |
| PA ,mean±SD | 5.22±1.13 | 4.31±0.97 | 5.35±1.09 | <0.001 |
| ECW(L) , median (IQR) | 12.75(10.6,14.7) | 11.25(10.6,14.43) | 12.85(10.75,14.73) | 0.320 |
| SLM（kg）, median (IQR) | 41.55(35.2,48.23) | 37.4(33.78,45.33) | 41.8(35.78,48.63) | 0.078 |
| WC(cm) , median (IQR) | 79.2(71.1,89) | 80.65(72.4,91.4) | 79.2(70.93,88.43) | 0.569 |
| VFA(cm^2^) , median (IQR) | 72.2(45.03,105.55) | 105.25(58.5,147.63) | 70(43.58,100.05) | 0.010 |
| LVEF: left ventricular ejection fraction; BP:blood pressure; BMI: Body Mass Index；ICW:Intracellular Water；TBW: Total Body Water；ECW:Extracellular Water；PBF:Percentage of Body Fat；SLM:Soft Lean Mass；WC: Waist Circumference ；VFA: Visceral Fat Area；PA:Phase Angle | | | | |

| **Table 5. Baseline characteristics of study population by Age.** | | | | |
| --- | --- | --- | --- | --- |
| **Characteristics** | Total | Young-group | Old-group | P |
|  |  | ≤60 years old | >60 years old |  |
|  | 158 | 88 | 70 |  |
| Gender，Female, N（%） | 63 (39.9) | 41(46.6) | 22(31.4) | 0.053 |
| Age(years) , median(IQR) | 59(49,66) | 50.5(40.3,56) | 68(63,73) | <0.001 |
| Dialysis vintage(months), median(IQR) | 59(44,82.8) | 63.5(46.3,84.3) | 54(43,82.8) | 0.225 |
| Vascular access,N(%) | 158 |  |  | 0.502 |
| Tunneled cuffed catheter | 41 (25.9) | 21(23.9) | 20(28.6) |  |
| Avf | 117 (74.1) | 67(76.1) | 50(71.4) |  |
| Dialysis prescription,N(%) | 158 |  |  | 0.085 |
| Twice a week | 35 (22.2) | 14(15.9) | 21(30) |  |
| Five times every 2 weeks | 21 (13.3) | 14(15.9) | 7(10) |  |
| Three times a week | 102 (64.6) | 60(68.2) | 42(60) |  |
| Hypertension，N（%） | 146 (92.4) | 80(90.9) | 66(94.3) | 0.622 |
| Diabetes，N（%） | 52 (32.9) | 21(23.9) | 31(44.3) | 0.007 |
| History of smoking，N（%） | 33 (20.9) | 16(18.2) | 17(24.3) | 0.348 |
| Ischemic heart disease, N (%) | 33(20.9) | 21(23.9) | 12(17.1) | 0.302 |
| LVEF,% | 60(30-65) | 60(33-65) | 58(30-65) | 0.471 |
| Hemoglobin(g/L),median(IQR) | 107(94,117) | 105.5(92,116.8) | 108.5(97,117) | 0.559 |
| Albumin(g/L),mean±SD | 38.3±3.3 | 39.0±3.5 | 37.3±2.9 | 0.001 |
| Urea nitrogen(mmol/L), mean±SD | 23.0±6.1 | 22.9±6.4 | 23.2±5.8 | 0.759 |
| Creatinine(umol/L),mean±SD | 897.6±256.6 | 933.0±270.0 | 853.1±233.0 | 0.052 |
| Phosphorus(mmol/L),mean±SD | 1.7±0.4 | 1.72±0.45 | 1.71±0.41 | 0.799 |
| Calcium(mmol/L),median(IQR) | 2.12(1.97,2.28) | 2.18(1.98,2.34) | 2.05(1.92,2.20) | 0.016 |
| PTH(pg/ml), median(IQR) | 397(264,604) | 416(189,661) | 388(279,563) | 0.840 |
| Uric acid(umol/L), median(IQR) | 444(358,514) | 442(347,513) | 445(371,519) | 0.518 |
| Triglyceride (mmol/L), median (IQR) | 1.28(0.98,1.94) | 1.27(0.95,2.01) | 1.29(0.98,1.83) | 0.967 |
| TC (mmol/L), median (IQR) | 3.63(3.08,4.41) | 3.57(3.11,4.42) | 3.72(2.99,4.41) | 0.827 |
| LDL(mmol/L),mean±SD | 2.06±0.71 | 1.97±0.63 | 2.16±0.77 | 0.110 |
| systolic BP(mmHg),mean±SD | 140±16 | 138±17 | 143±15 | 0.048 |
| diastolic BP(mmHg),mean±SD | 79±10 | 82±9 | 76±9 | <0.001 |
| pulse pressure(mmHg),mean±SD | 61±15 | 56±15 | 67±13 | <0.001 |
| SBPV(%),median (IQR) | 8.05(5.98,10.20) | 7.75(5.35,10.2) | 8.45(6.78,10.18) | 0.096 |
| DBPV(%),mean±SD | 7.63±2.56 | 7.49±2.53 | 7.82±2.59 | 0.429 |
| Antihypertensive drug,N(%) |  |  |  |  |
| ACEI | 62 (39.2) | 34(38.6) | 28(40) | 0.862 |
| ARB | 83 (52.5) | 46(52.3) | 37(52.9) | 0.942 |
| β-blocker | 139 (88) | 74(84.1) | 65(92.9) | 0.092 |
| CCB | 141 (89.2) | 76(86.4) | 65(92.9) | 0.191 |
| α-blockers | 51 (32.3) | 25(28.4) | 26(37.1) | 0.243 |
| BMI(kg/m^2^) ,mean±SD | 22.21±3.25 | 21.94±3.31 | 22.54±3.16 | 0.251 |
| ICW(L) ,mean±SD | 20.00±4.05 | 20.44±4.25 | 19.45±3.74 | 0.130 |
| TBW(L) ,mean±SD | 32.78±6.44 | 33.25±6.61 | 32.18±6.21 | 0.305 |
| PBF（%）,mean±SD | 26.17±10.11 | 24.00±9.46 | 28.89±10.31 | 0.002 |
| ECW/TBW,mean±SD | 0.39±0.01 | 0.39±0.01 | 0.40±0.01 | <0.001 |
| PA ,mean±SD | 5.22±1.13 | 5.51±1.21 | 4.85±0.91 | <0.001 |
| ECW(L) , median (IQR) | 12.75(10.6,14.7) | 13.00(10.60,14.65) | 12.65(10.95,14.83) | 0.770 |
| SLM（kg）, median (IQR) | 41.55(35.2,48.23) | 43.65(35.0,48.50) | 39.95(34.58,47.63) | 0.334 |
| WC(cm) , median (IQR) | 79.2(71.1,89) | 76.7(69.4,88.1) | 80.7(72,92.3) | 0.069 |
| VFA(cm^2^) , median (IQR) | 72.2(45.03,105.55) | 61.6(39.2,84.0) | 92.3(48.2,125.7) | <0.001 |
| LVEF: left ventricular ejection fraction; BP:blood pressure; BMI: Body Mass Index；ICW:Intracellular Water；TBW: Total Body Water； ECW:Extracellular Water；PBF:Percentage of Body Fat；SLM:Soft Lean Mass；WC: Waist Circumference；VFA: Visceral Fat Area；PA:Phase Angle | | | | |

| **Table6. Univariate Cox regression for all-Cause Mortality or cardiovascular (CV).** | | |
| --- | --- | --- |
| Variable | HR(95%CI) | p |
| age (years) | 1.080(1.051-1.111) | <0.001 |
| Gender (female as Ref) | 2.084(1.053-4.124) | 0.035 |
| diabetes(no=0,yes=1) | 2.946(1.611-5.385) | <0.001 |
| Albumin (g/L) | 0.845 (0.769-0.928) | <0.001 |
| DBPV (%) | 1.121(1.005-1.251) | 0.040 |
| systolic BP (mmHg) | 1.013(0.994-1.032) | 0.173 |
| diastolic BP (mmHg) | 0.968(0.940-0.997) | 0.028 |
| pulse pressure (mmHg) | 1.025(1.008-1.042) | 0.004 |
| Phosphorus (mmol/L) | 0.402(0.195-0.830) | 0.014 |
| ECW/TBW | 1.517E+19(4.736 E+10-4.857 E+27) | <0.001 |
| Phase Angle | 0.555(0.422-0.729) | <0.001 |
| DBPV: dystolic blood pressure variability; BP: blood pressure;TBW: Total Body Water; ECW:Extracellular Water. | | |
